# Destination Therapy with the magnetically levitated HVAD

**DOI:** 10.1186/1749-8090-10-S1-A287

**Published:** 2015-12-16

**Authors:** Christian Heim, Johannes Rösch, Thorsten Zielezinski, Fatos Ballazhi, Markus Kondruweit, Michael Weyand, Rene Tandler

**Affiliations:** 1 Department of Cardiac Surgery, Friedrich-Alexander University, Erlangen, Germany, 91054

## Background/Introduction

Destination therapy (DT) with mechanical circulatory support (MCS) for advanced-stage heart failure patients is an alternative therapy when there is no option of eventually providing the patient with heart transplantation. New generation magnetically levitated pumps encourage a broader use of DT.

## Aims/Objectives

Aim of this study was to analyze follow up complications and survival after DT with MCS.

## Method

13 consecutive patients at our hospital were included in this study. Only adult patients with magnetically levitated Heartware^® ^LVADs were included in this study. We analyzed the indication and outcome of LVAD destination therapy assessing risk profile, INTERMACS classification, complication rates, and survival after LVAD implantation (cut-off 30/06/2014).

## Results

Our data show preoperative values including age [67.1 y+5.0 y] and INTERMACS classification [range 2-4]. The indication for DT includes old age, compliance problems, and severe comorbidities. Outcome data demonstrate a low 30-day mortality in elective DT patients (100% survival). Mean follow up after permanent Heartware implantation was in average 544 days (range, 160-1512). 1 year survival after DT with HeartWare was 83.3%. Most frequent complications include bleeding and stroke (1 GI-bleeding, 1 permanent stroke, 1 recovered from SAB, 1 sternal wound infection). Using Heartware as DT LVAD lead to reduction of required INR and hereby reduction of complication rate.

## Discussion/Conclusion

Destination therapy with magnetically levitated HeartWare LVAD is associated with lower in-hospital mortality compared to heart transplant patients. Long-term survival after destination therapy is possible. Especially new magnetically levitated pumps with lower INR required might help to further reduce LVAD complications

